# Expression of microRNAs in Horse Plasma and Their Characteristic Nucleotide Composition

**DOI:** 10.1371/journal.pone.0146374

**Published:** 2016-01-05

**Authors:** Seungwoo Lee, Seungwoo Hwang, Hee Jeong Yu, Dayoung Oh, Yu Jung Choi, Myung-Chul Kim, Yongbaek Kim, Doug-Young Ryu

**Affiliations:** 1 BK21 Plus Program for Creative Veterinary Science Research, Research Institute for Veterinary Science and College of Veterinary Medicine, Seoul National University, 1 Gwanak-ro, Gwanak-gu, Seoul, 08826, South Korea; 2 Korean Bioinformation Center, Korea Research Institute of Bioscience and Biotechnology, Daejeon, 34141, South Korea; Cankiri Karatekin University, TURKEY

## Abstract

MicroRNAs (miRNAs) in blood plasma are stable under high levels of ribonuclease activity and could function in tissue-to-tissue communication, suggesting that they may have distinctive structural characteristics compared with non-circulating miRNAs. In this study, the expression of miRNAs in horse plasma and their characteristic nucleotide composition were examined and compared with non-plasma miRNAs. Highly expressed plasma miRNA species were not part of the abundant group of miRNAs in non-plasma tissues, except for the eca-let-7 family. eca-miR-486-5p, -92a, and -21 were among the most abundant plasma miRNAs, and their human orthologs also belong to the most abundant group of miRNAs in human plasma. Uracil and guanine were the most common nucleotides of both plasma and non-plasma miRNAs. Cytosine was the least common in plasma and non-plasma miRNAs, although levels were higher in plasma miRNAs. Plasma miRNAs also showed higher expression levels of miRNAs containing adenine and cytosine repeats, compared with non-plasma miRNAs. These observations indicate that miRNAs in the plasma have a unique nucleotide composition.

## Introduction

MicroRNAs (miRNAs) represent a class of small (~22 nt) noncoding RNAs, which regulate gene expression by binding to specific mRNA targets and promoting their degradation and/or translational inhibition [1, 2]. miRNAs recognize many mRNAs with partial complementarity, mostly involving a “seed” region that encompasses residues 2–8 from the 5′-end, or 5′2–5′8.

The genome of the horse (*Equus caballus*) has 64 chromosomes (2n = 64) containing 2.7 Gb with 20,000 protein-coding genes [3]. To date, 690 mature horse miRNA species have been identified (http://www.mirbase.org/, accessed May 2015) in various tissues including the liver, colon, muscle, cartilage, subchondral bone, and ovarian follicle as well as cord blood-derived mesenchymal stromal cells [4–8].

Uracil and cytosine are typically over- and under-represented in mammalian miRNAs, respectively [9–13]. Analyses of position-dependent nucleotide composition have demonstrated that uracil predominates at both ends of mammalian miRNAs and at the 5′9 position [9–13], while depletions of guanine and cytosine nucleotides are observed at 5′- and 3′- ends of miRNAs, respectively [9, 13]. However, the guanine–cytosine content of the seed region is higher than those of other regions [14].

Many studies have detected miRNAs in a variety of extracellular biological fluids such as plasma or serum, which contain miRNAs mostly derived from blood and endothelial cells [15–17]. Tissue-specific miRNAs for example, from the liver, are also represented, indicating that circulating miRNAs have various origins [18–21]. Although the biological function and origin of plasma miRNAs remain to be determined, some plasma miRNA species could be involved in inter-cellular and inter-tissue signal communication [22]. Circulating miRNAs in the plasma are protected from degradation despite the presence of high ribonuclease activities [16, 23, 24].

We hypothesized that plasma miRNAs may have unique structural characteristics compared with intracellular miRNAs, such as nucleotide composition, to ensure stability and function in their distinctive environment. To investigate this, we examined the expression and nucleotide composition of plasma miRNAs in the horse, and compared them with those of other horse tissues.

## Materials and Methods

### Horses

All animal experiments were approved by the Institutional Animal Care and Use Committees of Seoul National University (Approval #SNU–131218–1) and performed in accordance with institutional guidelines on animal use in research and with the EU Directive 2010/63/EU for animal experiments. Three healthy geldings, 21–25 years of age, were fasted in individual stalls without exercise for 12 h. Blood samples were then collected from the jugular vein into conical tubes containing ethylenediaminetetraacetic acid (Sigma-Aldrich, St. Louis, MO). Immediately after collection, each blood sample was gently inverted several times and placed on ice. Plasma was separated by centrifuging at 1500 ×*g* for 20 min and stored at −80°C until further use.

### Library preparation and sequencing

Total RNA was extracted from plasma using Trizol reagent (Invitrogen, Carlsbad, CA) according to the manufacturer’s instructions. Residual DNA was removed with DNase I (Takara Bio, Shiga, Japan) using the manufacturer’s guidelines. The RNA integrity number was used to assess integrity with the Agilent 2100 Bioanalyzer (Agilent Technologies, Palo Alto, CA). Total RNA with an RIN value greater than 7 was separated on a 15% denaturing polyacrylamide gel, and small RNAs ranging from 18 to 32 nucleotides were gel purified.

The small RNA library was prepared with the TruSeq Small RNA Sample Prep Kit (Illumina, San Diego, CA) according to the manufacturer’s instructions. Briefly, the adapters (5′ adapter, 5′-GUUCAGAGUUCUACAGUCCGACGAUC-3′ and 3′ adapter, 5′-UCGUAUGCCGUCUUCUGCUUGUidT-3′) were ligated to both ends of the purified small RNAs and a reverse-transcription reaction was performed to generate single-stranded cDNA using the primer 5′-CAAGCAGAAGACGGCATACGA-3′. cDNA was then PCR amplified using the primer pair forward primer, 5′-AATGATACGGCGACCACCGACAGGTTCAGAGTTCTACAGTCCGA-3′ and reverse primer, 5′-CAAGCAGAAGACGGCATACGA-3′. The amplified cDNA constructs were gel-purified and then sequenced on the HiSeq2000 (Illumina) according to the manufacturer's instructions, using the sequencing primer 5′-CGACAGGTTCAGAGTTCTACAGTCCGACGATC-3′.

### Sequencing data processing

Small RNA sequences were analyzed using the CLC Genomics Workbench version 6.5.1 (CLC bio, Aarhus, Denmark). Briefly, small RNA sequences were filtered for quality and size, and reads of low quality and lengths less than 15 were discarded. Clean reads were mapped to precursor miRNA sequences from mirbase release 21 (http://www.mirbase.org/) and noncoding RNA sequences from Ensembl horse genome release 78 (http://www.ensembl.org/), allowing two mismatches. miRNA variants were determined based on the presence of up to five additional nucleotides on either the 5′ or 3′ end of the reads.

### Data access

Raw sequence datasets in FASTQ format were deposited in the NCBI Sequence Reads Archive under accession numbers SRX170338, SRX170339, and SRX170340. MiRNA sequence data for non-plasma tissues are from Kim *et al*. [8]. The accession numbers for the colon tissues are SRX187171, SRX187172, SRX187173, and SRX187174; those for the muscle tissues are SRX187166, SRX187167, SRX187168, and SRX187169; those for the liver tissues are SRX187162, SRX187163, SRX187164, and SRX187165.

### Normalization of miRNA expression levels

Read count data were normalized by the trimmed mean of M-values (TMM) method [25] available in the edgeR Bioconductor package [26]. The TMM normalization method can adjust for situations where different tissues express diverse miRNA repertoires such that some miRNA genes may be very highly expressed in one tissue, but not in another. The TMM-normalized expression values were thus obtained as count per million (CPM) unit. Subsequent to normalization, we filtered out miRNAs expressed at very low levels. Because the smallest group size in the plasma is three samples, we retained those miRNAs with at least one CPM in at least three samples. This low expression filtering retained 266 miRNAs out of a total of 428.

### Hierarchical clustering

For unsupervised hierarchical clustering analysis, logarithms of CPM values of the 266 miRNAs were taken and inputted to the EMA Bioconductor package [27] using the average linkage method with a Pearson centered correlation as a similarity metric. To draw a heatmap, log CPM values of each miRNA were standardized across samples by z-transformation such that the row mean and variance became 0 and 1, respectively.

### Identification of potential novel miRNAs

The reads that did not match any database were mapped to the horse reference genome (EquCab2) using SOAP version 1.11 [28], with no mismatches allowed. The reads with perfect matches were subjected to Mireap version 0.2 (http://sourceforge.net/projects/mireap/) to predict novel miRNAs. The precursors of predicted novel miRNAs were further subjected to Mipred [29] to filter out pseudo-miRNAs as previously described [30].

### Target prediction

The 5′ and 3′ untranslated regions of horse genes were extracted from the Ensembl horse genome release 78 (http://www.ensembl.org/). A search for miRNA target genes was performed using Target-align [31] with the following parameters [32–34]: (1) no more than four mismatches between the small RNA and its target (G–U nucleotides count as 0.5 mismatches); (2) no more than two adjacent mismatches in the miRNA/target duplex; (3) no mismatches in positions 10 and 11 of the miRNA/target duplex; and (4) no more than 2.5 mismatches in positions 1 and 12 of the miRNA/target duplex (5′ of miRNA). Functional annotation of the target genes was performed using the PANTHER classification system version 9.0 (http://www.pantherdb.org/) [35].

### Nucleotide composition

miRNA sequences were imported from the CLC Genomics Workbench into Excel 2013 (Microsoft, Redmond, WA), and were arranged such that each spreadsheet cell contained a nucleotide for a specific miRNA position. The COUNTIF and PRODUCT commands as well as the Pivot Table feature were used to determine the nucleotide compositions. The number of 22 nt-miRNAs containing at least one MNR (four nucleotides or longer) for each nucleotide was estimated using a Perl script (S1 Material).

### Statistical analyses

Statistical analyses were performed using SPSS 15.0 (SPSS, Chicago, IL). The comparison of miRNA expression levels was evaluated using one-way analysis of variance (ANOVA) with Tukey’s *post hoc* test. The comparison of nucleotide compositions was performed using one-way ANOVA with Tukey’s *post hoc* test and the Student's *t*-test. Data were considered significantly different when *P* was < 0.05.

## Results

### MiRNA species enriched in blood plasma

Next generation sequencing-based small RNA profiles were generated for three horse plasma libraries (Table 1), with 21.8–25.6 million sequencing reads obtained from each library. After removing low-quality reads from the raw sequences, we obtained 21.7–25.4 million clean reads, of which 10.3–15.8 million (45.3–72.7%) were previously known miRNA species.

**Table 1:** Sequencing read statistics for small RNA libraries of horse plasma.

Type	Library #1		Library #2		Library #3	
	Count	Percent (%)	Count	Percent (%)	Count	Percent (%)
**Total reads**	21,754,488	100	22,626,015	100	25,555,484	100
**Contaminant reads**	63,730	0.29	236,244	1.04	152,460	0.60
**Clean reads**	21,690,758	99.71	22,389,771	98.96	25,403,024	99.40
** - miRNA reads**	15,822,246	72.73	10,250,013	45.30	14,868,199	58.18
** - Other non-coding RNA reads**	3,251,323	14.95	10,198,778	45.08	7,889,233	30.87
** - Unannotated reads**	2,617,189	12.03	1,940,980	8.58	2,645,592	10.35

**Fig 1:**
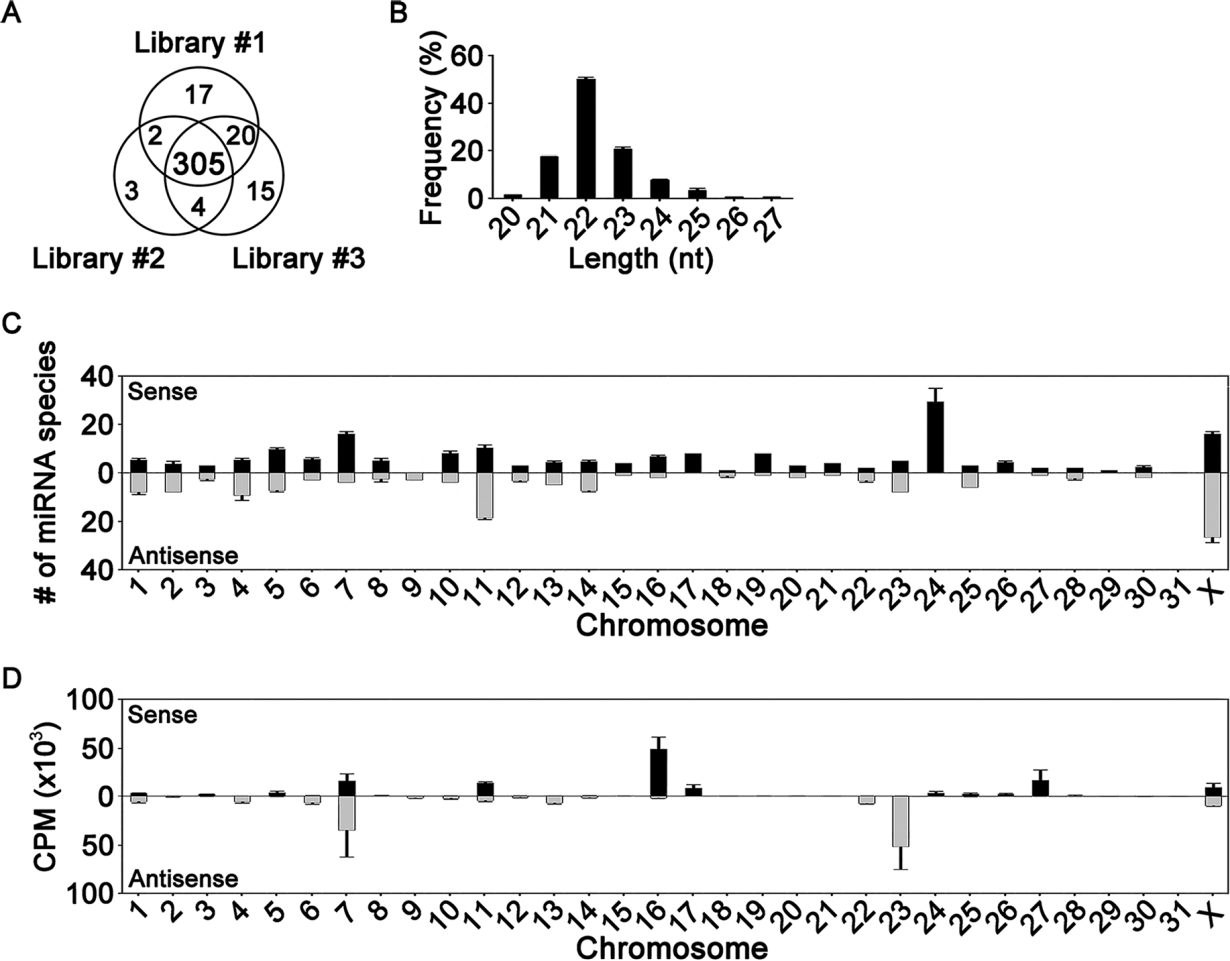
Sequencing of miRNAs in three horse plasma libraries. (A) Venn diagram representing the numbers of miRNA species identified in the libraries. (B) Length distribution of miRNAs. The x-axis represents sequence lengths, and the y-axis the occurrence frequency of miRNA species for each given length. (C, D) Chromosomal location of miRNA genes. The x-axes represent chromosome number, and the y-axes the numbers of miRNA species (C) and reads (D) on the respective chromosome. Black and gray bars represent sense and antisense strands, respectively. For (C) and (D), data are shown as mean ± S.D. (n = 3).

Sequencing identified 366 unique miRNA species in the three libraries, of which 305 were commonly found (Fig 1A). The sequences were 20–27 nt in length, and those 22 nt long were the most frequent (Fig 1B). The genes for the 366 miRNA species are located within all of the horse chromosomes, with the exception of the smallest chromosome 31 (Fig 1C and 1D). Among these, chromosomes 11, 24, and X contained the highest number of genes for plasma miRNAs. Chromosomes 7, 16, and 23 contributed most to the plasma miRNA content, as assessed by the sum of the CPM levels for each miRNA species.

Ten plasma miRNA species exhibiting the highest expression levels in the libraries are listed with their characteristics in Table 2. These accounted for 68.6% of total plasma miRNA reads, and the three most highly expressed miRNA species, eca-let-7f, -7a, and eca-miR-191a, accounted for 40.6%. Those reads that had not been annotated were used to identify potentially novel miRNA species *in silico*. S1 Table lists the five potentially novel miRNA species exhibiting the highest expression levels.

**Table 2:** Ten most abundant miRNA species in horse plasma. Location represents the chromosome on which the miRNA genes are located, start and end sites, and either sense [+] or antisense strand [−].

**miRNA**	**Mature sequence**	**Location**	**Potential target genes**	**CPM per total CPM (%)**
eca-let-7f	UGAGGUAGUAGAUUGUAUAGUU	Chr23: 54150758–54150844 [–]	PKD1L3, SETD9, SOS2, SRPK2, WAC	16.9
eca-let-7a	UGAGGUAGUAGGUUGUAUAGUU	Chr7: 29543908–29543979 [–]	WAC	13.2
eca-miR-191a	CAACGGAAUCCCAAAAGCAGCUG	Chr16: 38001159–38001232 [+]	IL13RA1, TRPM3	10.5
eca-let-7g	UGAGGUAGUAGUUUGUACAGUU	Chr16: 35442180–35442267 [+]	ACSM3, BTRC, CDH7, RIMS2, WAC, WDR20, ZSWIM5	7.58
eca-miR-486-5p	UCCUGUACUGAGCUGCCCCGAG	Chr27: 3709569–3709634 [+]	Not Found	5.90
eca-miR-24	UGGCUCAGUUCAGCAGGAACAG	Chr7: 44930163–44930230 [+]	AHDC1, AVEN, DCAF10, KANSL3, OTOP1	3.16
eca-miR-223	UGUCAGUUUGUCAAAUACCCCA	ChrX: 48492588–48492691 [+]	Not Found	3.07
eca-miR-21	UAGCUUAUCAGACUGAUGUUGA	Chr11: 33863745–33863816 [+]	ESR1, SMARCA2, ZNF800	2.91
eca-miR-92a	UAUUGCACUUGUCCCGGCCUGU	Chr17: 61793120–61793180 [+]	STT3A	2.87
eca-miR-103	AGCAGCAUUGUACAGGGCUAUGA	Chr14: 12370468–12370539 [+]	Not Found	2.52
Total				68.6

### Clustering of miRNA expression

**Fig 2:**
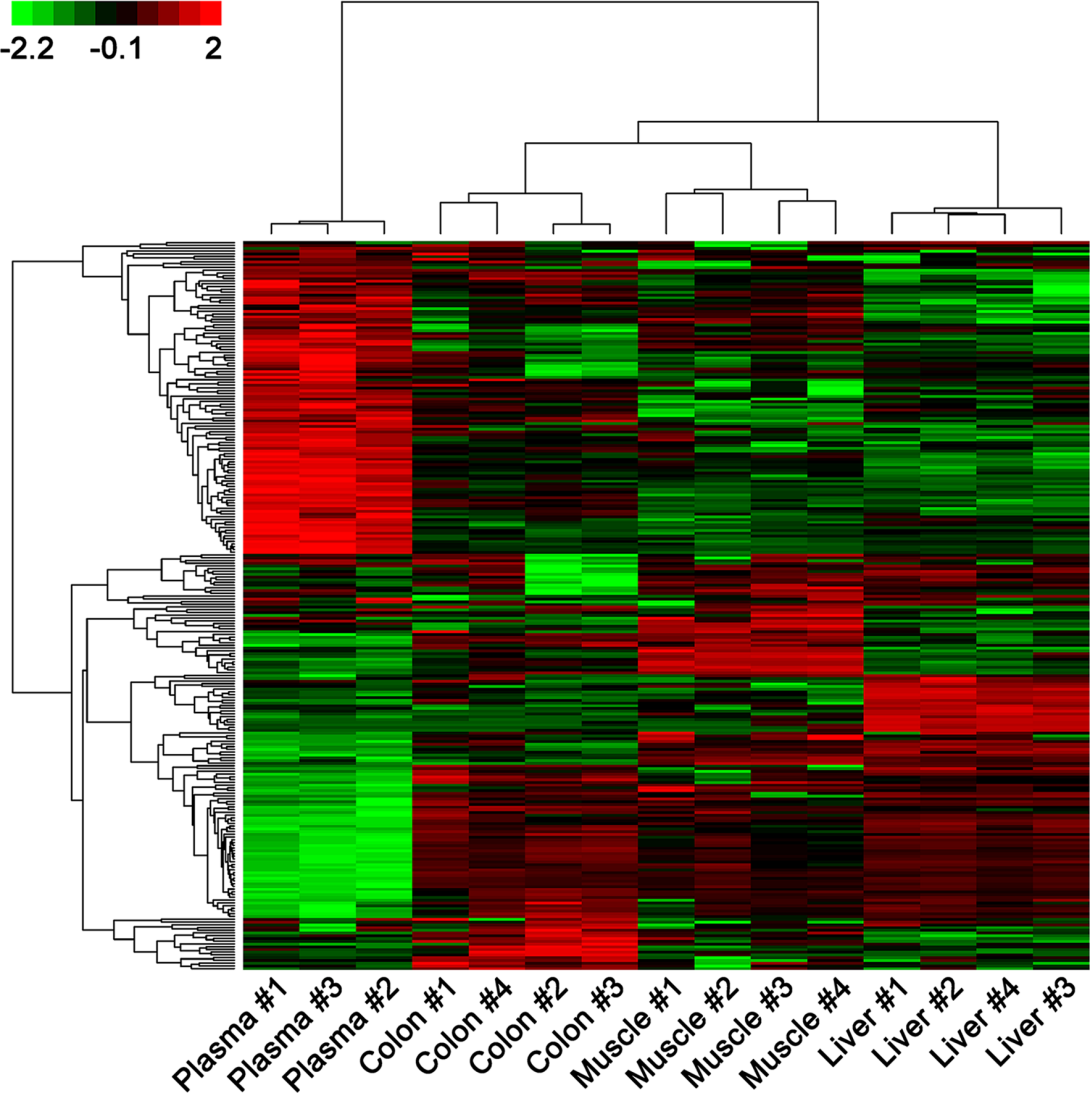
Unsupervised hierarchical clustering of horse miRNA expression profiles. Values in the color key represent z-transformed expression values, such that highest expression values of each row correspond to bright red, and the lowest to bright green. Plasma samples constitute a distinct cluster, separated from the other three tissue clusters.

As shown in the dendrogram at the top of Fig 2, samples were clustered according to tissue origins. Plasma samples constituted a distinct cluster, separated from the other three tissue clusters by a large inter-cluster branch length [8]. The plasma cluster also showed the smallest intra-cluster branch length reflecting the homogeneous expression profiles of the plasma samples. Two large miRNA clusters were observed in the gene dendrogram: the first consisted of miRNAs with consistently high plasma expression in plasma, and showed a simple dendrogram structure. The second cluster consisted of miRNAs with lower plasma expression, and showed a more complicated dendrogram structure with several subclusters, reflecting the heterogeneous expression profiles between the subclusters. A total of 87 plasma miRNAs exhibited more than 2-fold higher expression levels than each of the three non-plasma tissues, and 30 exhibited more than 10-fold higher expression levels; 81 showed more than 2-fold lower expression levels and 50 more than 10-fold lower expression levels. MiRNA species that showed higher and lower levels of expression (CPM) in the plasma compared with the liver, colon, and muscle tissues are listed in S2 and S3 Tables, respectively.

### Expression of blood plasma-enriched miRNA species in non-plasma tissues

**Fig 3:**
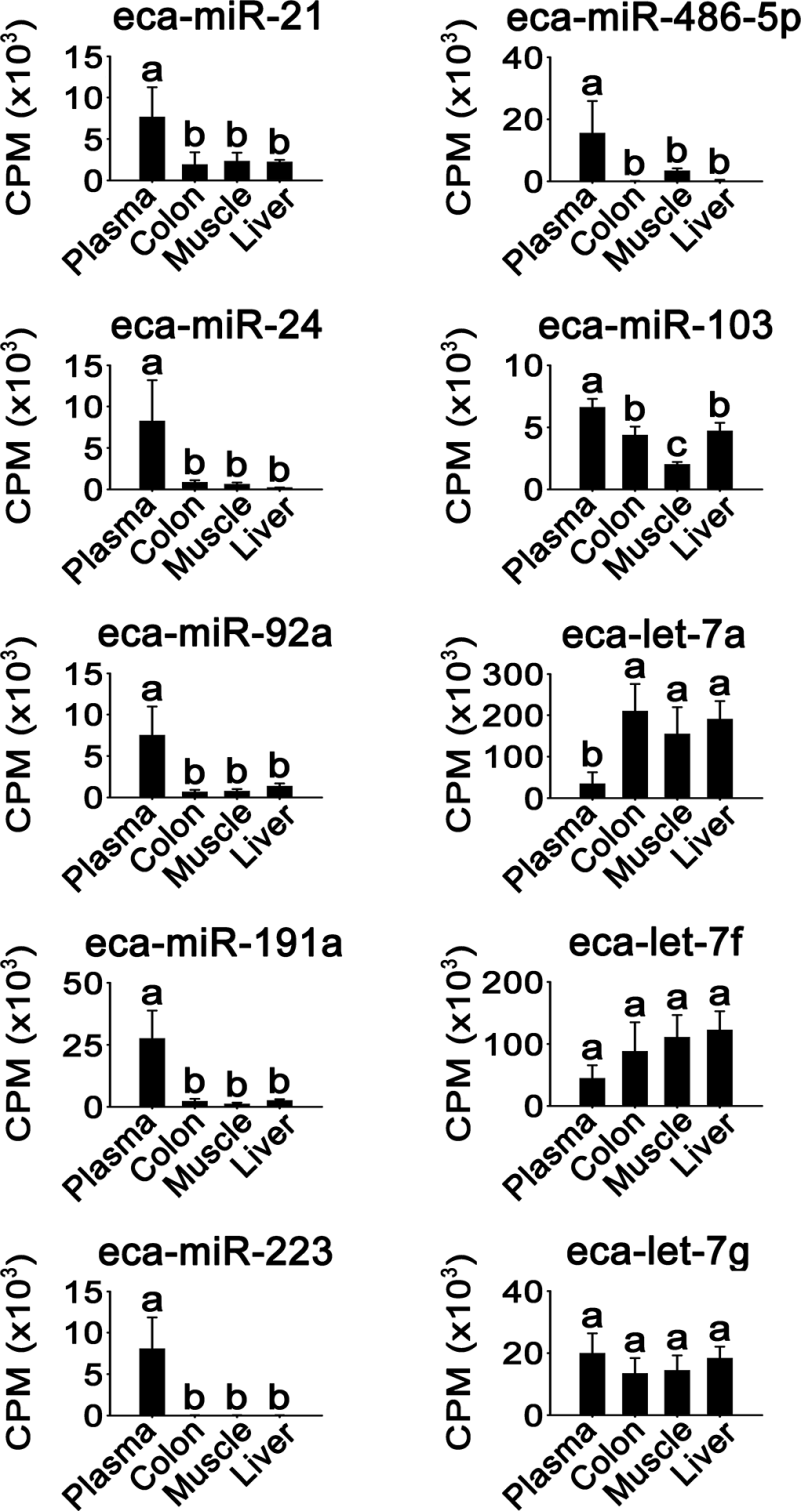
Differential expression. Tissue distribution was examined for 10 miRNA species that were most highly expressed in horse plasma. Different alphabetical letters (a, b, and c) above the bars represent significant differences between the tissues (n = 3–4, one-way ANOVA with Tukey’s *post hoc* test, *P* < 0.05).

The expression of the 10 miRNA species exhibiting the highest plasma expression (Table 2) was next examined in the colon, muscle, and liver (Fig 3). Seven miRNA species, eca-miR-21, -24, -92a, -191a, -223, -486-5p, and -103, were found to have significantly higher expression levels in the plasma than the three other tissues (*P* < 0.05). The expression levels for eca-let-7f and -7g did not differ significantly among the four tissue types, whereas eca-let-7a expression was significantly lower in the plasma (*P <* 0.05). This suggests that the 10 most abundant plasma miRNA species did not belong to the abundant miRNA group in non-plasma tissues, except for the eca-let-7 family species (S4 Table).

### MiRNA target prediction and gene ontology (GO) analyses

To examine the functional importance of plasma miRNAs, we derived potentially affected targets using a prediction program. A total of 22 potential target mRNAs were identified for 10 miRNAs having the highest expression in the plasma (Table 2). WW domain containing adaptor with coiled-coil (*WAC*) mRNA was shown to be a putative common target of three of the miRNA species with nearly identical sequences: eca-let-7a, -7f, and -7g. No potential target was identified for eca-miR-486-5p, -223, or -103.

Among the potential targets, 10 mRNAs were subcategorized into 17 GO classifications including nine biological processes (S1A Fig), six molecular functions (S1B Fig), and two cellular components (data not shown). Specifically, “metabolic process” (seven targets, 23.3%) and “cellular process” (seven targets, 23.3%) were highly represented in the biological process of classification; together with “catalytic activity” (six targets, 31.6%) and “binding” (six targets, 31.6%) in the molecular function classification; and “cell part” (one target, 50%) and “membrane” (one target, 50%) in the cellular component classification.

### Overall nucleotide composition of miRNAs

**Fig 4:**
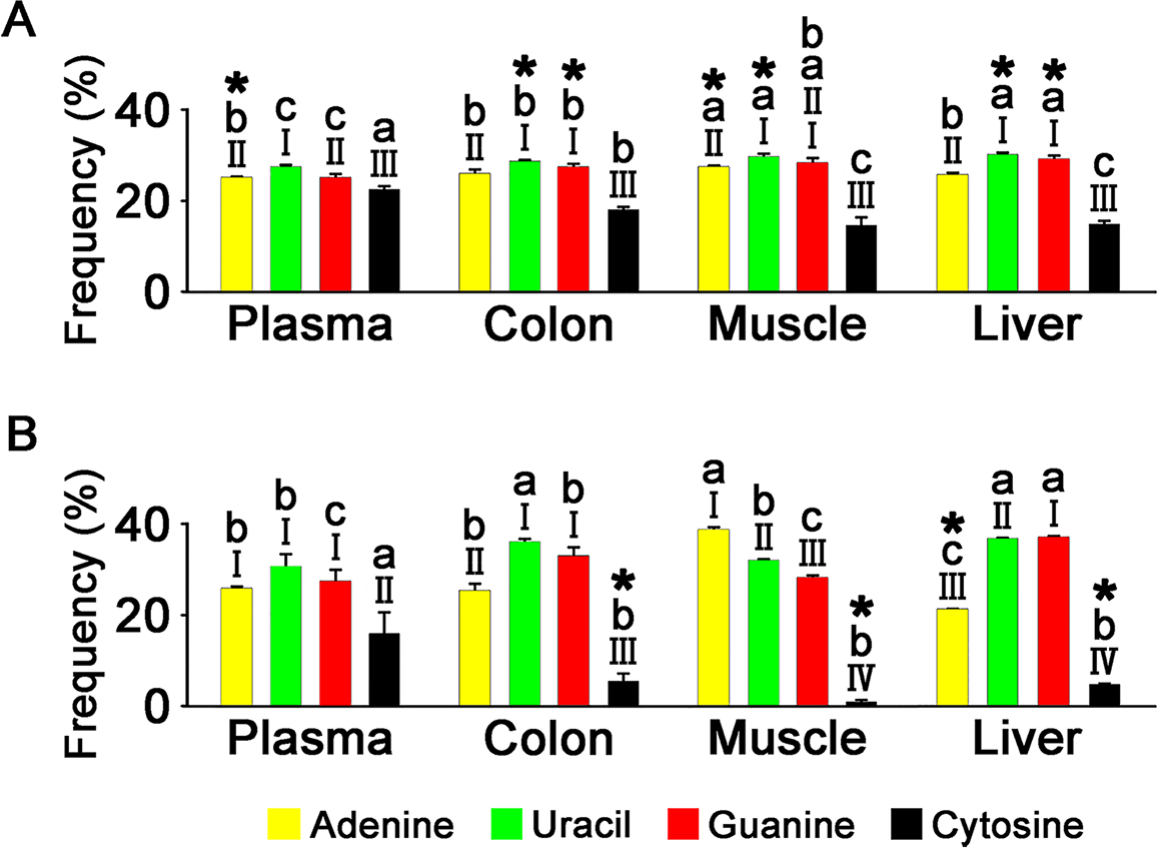
Overall nucleotide composition of miRNAs in different tissues, as assessed by miRNA expression-unweighted (A) and -weighted criteria (B). Different Roman numerals (I, II, III, and IV) above the bars represent significant differences between occurrence frequencies of nucleotides within the tissue, and different alphabetical letters (a, b, and c) represent the significant difference between occurrence frequencies of a nucleotide among the different tissues (n = 3–4, one-way ANOVA with Tukey’s *post hoc* test, *P* < 0.05). * indicates that the occurrence frequency of a nucleotide is smaller in the expression-unweighted criterion than -weighted counterpart within the tissue, or vice versa (Student’s *t*-test, *P* < 0.05).

We examined the overall nucleotide composition of miRNA in the four tissues, focusing particularly on cytosine frequencies. We conducted miRNA expression-unweighted (Fig 4A) and -weighted (Fig 4B) analyses, with the latter assessing the miRNA nucleotide composition by taking into account their expression levels in the tissues. Thus, miRNA species having higher expression levels would affect the nucleotide compositions to a greater extent in expression-weighted analysis.

In all four tissue miRNAs, cytosine was the nucleotide with the lowest frequency under both expression-unweighted and -weighted criteria (*P* < 0.05). Cytosine frequencies in non-plasma tissue miRNAs ranged from 51.2 to 65.7% and from 2.73 to 17.6% of the means of the frequencies of the other three nucleotides under expression-unweighted and -weighted criteria, respectively. Those equivalents in plasma miRNAs were much higher, at 86.5% and 56.8%, respectively.

The cytosine frequency was significantly higher in plasma miRNAs than in those of non-plasma tissues, at more than 1.25- and 2.88-fold under expression-unweighted and -weighted criteria, respectively (*P* < 0.05). Despite their high cytosine content, plasma miRNAs had a guanine–cytosine content slightly higher or similar to those of other tissue miRNAs, ranging from 1.05- to 1.11-fold and from 1.04- to 1.49-fold that of plasma miRNAs in the expression-unweighted and -weighted analyses, respectively. This may reflect the low guanine content of plasma miRNAs among the tissue miRNAs. The guanine content of plasma miRNAs was 86.1–92.6% of those of non-plasma tissues in expression-unweighted analysis (*P* < 0.05). In expression-weighted analysis, the guanine content of plasma miRNA was 73.9% and 83.1% of those of colon and liver miRNAs, respectively (*P* < 0.05), and not significantly different from that of muscle miRNAs.

In non-plasma tissue miRNAs, cytosine frequencies under expression-weighted analyses were lower by at least 3.17-fold than those under expression-unweighted analyses (*P* < 0.05). However, the difference in cytosine frequencies of plasma miRNAs was not significant between the two criteria.

### Position-dependent nucleotide composition of miRNAs

**Fig 5:**
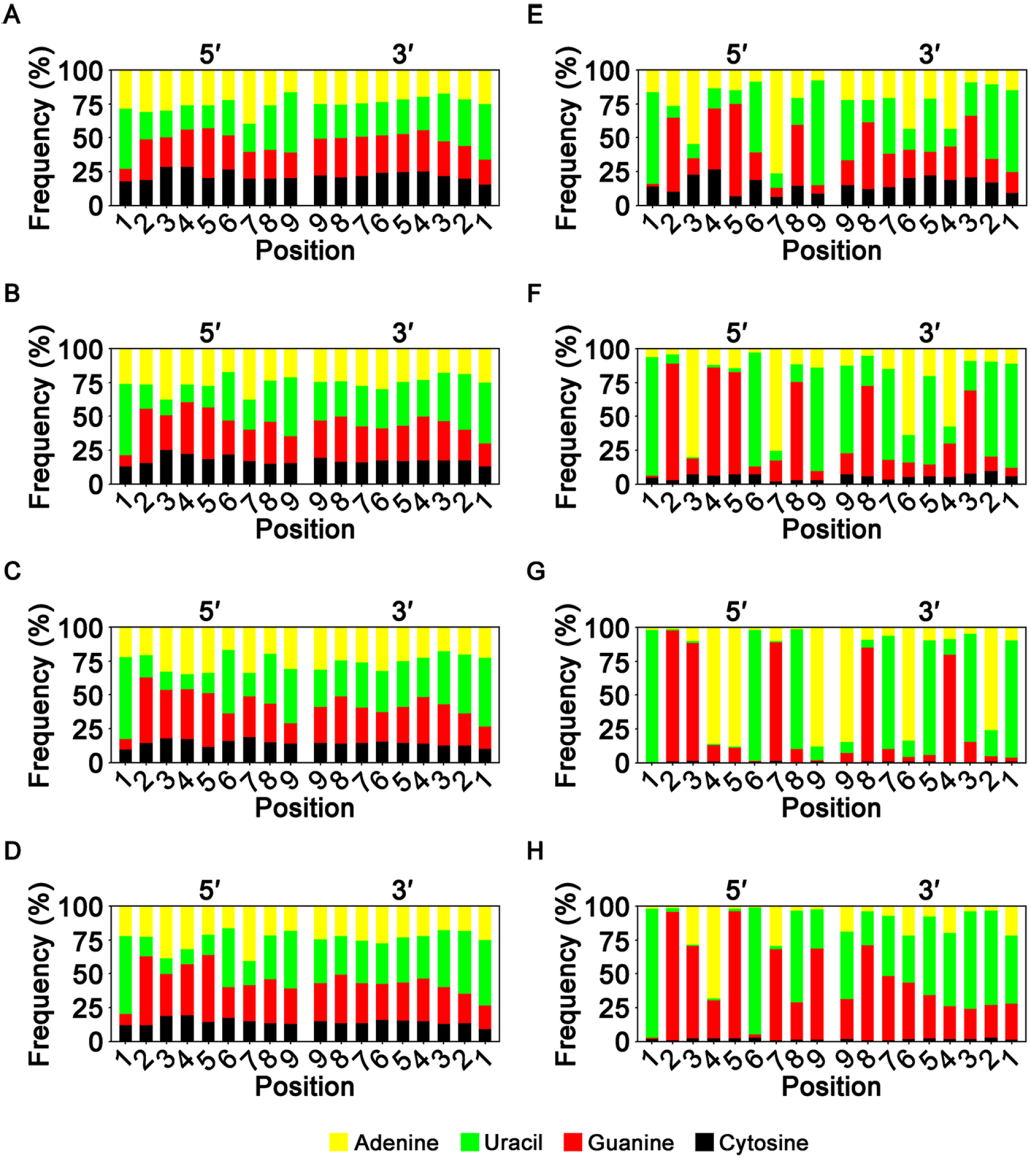
Position-dependent nucleotide composition for 5′- and 3′-ends of miRNAs. The composition was assessed in the expression-unweighted (A–D) and -weighted criteria (E–H), in plasma (A and E), colon (B and F), muscle (C and G), and liver (D and H). The x-axes represent nucleotide positions of miRNA sequences, and the y-axes the occurrence frequencies of nucleotides (n = 3–4).

Position-dependent nucleotide compositions were analyzed for 18 nucleotide positions of miRNAs, including the first to ninth nucleotide positions at both 5′- and 3′-ends (Fig 5). This aimed to assess nucleotide positions responsible for the low cytosine content in tissue miRNAs and the relatively high cytosine content in plasma miRNAs (Fig 4).

In expression-unweighted analysis, plasma miRNAs had the highest cytosine frequencies at 14 of the 18 nucleotide positions compared with the three other tissue types (*P* < 0.05; Fig 5A). The four exceptions were observed at 5′3, 5′5, 5′7, and 3′2. The cytosine frequency ranged from 15.6 to 28.5% at the 18 positions of plasma miRNA, with equivalents of 13.2–25.1%, 9.73–19.1%, and 9.44–19.4% for colon, muscle, and liver tissue miRNAs, respectively. The lowest and highest cytosine frequencies in both plasma and colon miRNAs were observed at 3′1 and 5′3, respectively, while these positions were 5′1 and 5′7 for muscle miRNAs, respectively, and 3′1 and 5′3 for the second lowest and highest cytosine frequencies in muscle miRNAs, respectively. In the liver miRNAs, 3′1 and 5′4 had the lowest and highest cytosine frequencies, respectively, while 5′3 had the second highest frequency.

In expression-weighted analysis, plasma miRNAs exhibited the highest cytosine frequencies at 16 of the 18 nucleotide positions compared with other tissues (*P* < 0.05; Fig 5E). The exceptions were observed at positions 5′5 and 3′1. In plasma miRNAs, cytosine occurred at frequencies of 6.2–26.4% in the 18 positions, with 5′7 and 5′4 having the lowest and highest frequencies, respectively. In the miRNAs of colon, muscle, and liver tissues, the lowest cytosine frequencies were found at 5′7, 5′1, and 5′2, where the frequencies were 2.2%, 0.34%, and 0.93%, respectively. The highest cytosine frequencies were found at 3′2, 5′3, and 3′2, where the frequencies were 9.61%, 1.50%, and 2.79%, respectively.

Variations of position-dependent cytosine frequencies were higher in the expression-weighted analysis than in the expression-unweighted analysis. The coefficients of variation (CVs) for cytosine frequencies among the 18 positions were 15.9–17.6% among the tissue miRNAs in the expression-unweighted analysis, compared with 30.1–37.7% in the expression-weighted analysis.

The other three nucleotides exhibited much higher variations in position-dependent nucleotide frequencies than cytosine. The CV ranges for adenine, uracil, and guanine frequencies at the 18 positions of the four tissue miRNAs were 21.1–27.1%, 31.2–43.6%, and 24.7–35.0% in the expression-unweighted analyses, respectively. The equivalents for the expression-weighted criterion were 73.5–122%, 69.8–108%, and 65.6–131%, respectively.

The position-dependent guanine–cytosine content was significantly higher at seven positions in the plasma miRNAs than those of other tissues in the expression-unweighted analysis: 5′1, 3′1, 3′2, and 3′4–3′7 (*P* < 0.05). The content differences between those of plasma and other tissue miRNAs were 1.54-fold or less. In the expression-weighted analysis, positions 5′1 and 5′6 had at least 2.5- and 3.0-fold higher guanine–cytosine contents in the plasma miRNAs than in other tissue miRNAs, respectively (*P* < 0.05). However, plasma miRNAs had at least 27.0% and 13.8% lower guanine–cytosine contents at positions 5′2 and 3′8 than the other tissue miRNAs, respectively (*P* < 0.05).

### Occurrence of mononucleotide repeat (MNR) motifs in miRNAs

**Fig 6:**
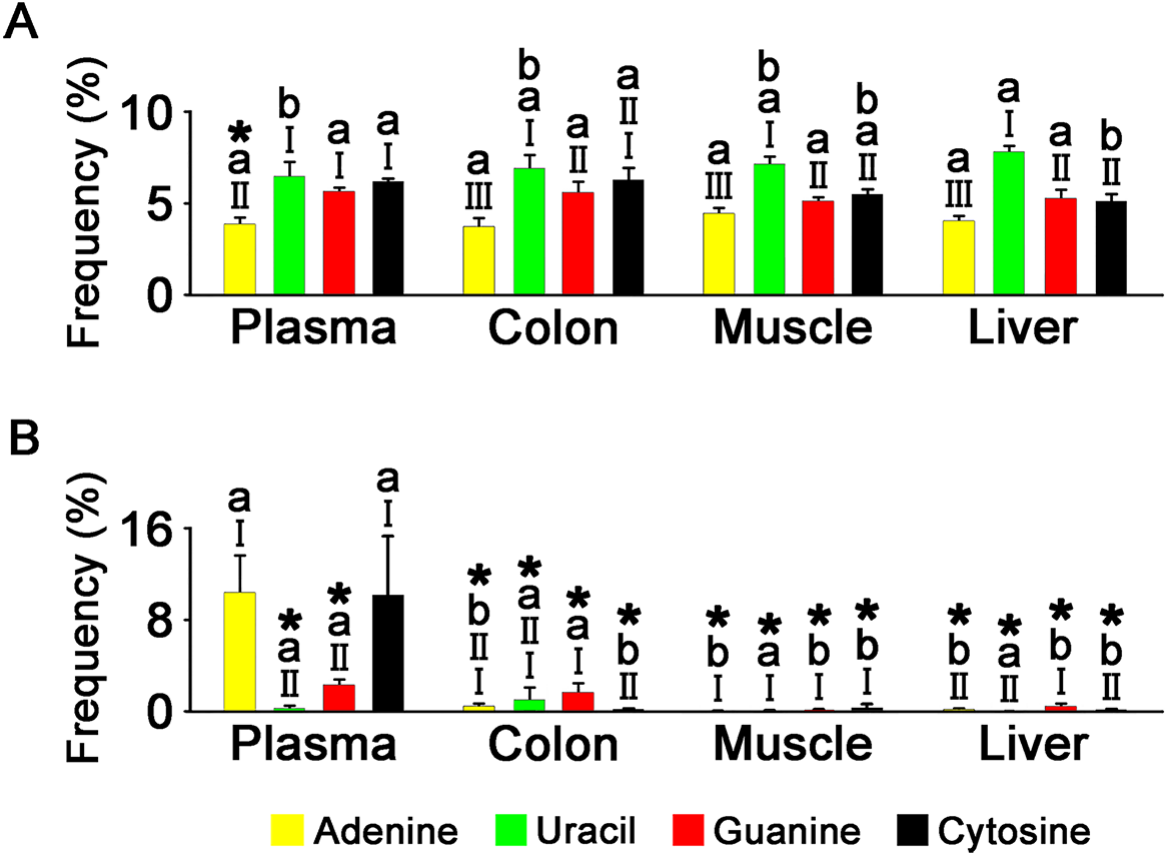
Occurrence frequencies of MNR-containing miRNAs (four repeats or longer) in tissue miRNAs, as assessed in expression-unweighted (A) and -weighted criteria (B). Different Roman numerals (I, II, and III) above the bars represent significant differences between frequencies of MNR-containing miRNAs within miRNAs of a tissue, and different alphabetical letters (a and b) represent the significant difference between frequencies of MNR-containing miRNAs for a nucleotide among different tissue miRNAs (n = 3–4, one-way ANOVA with Tukey’s *post hoc* test, *P* < 0.05). * indicates that the frequency of MNR-containing miRNAs for a nucleotide is smaller in the expression-unweighted criterion than the -weighted counterpart within the tissue miRNAs, or vice versa (Student’s *t*-test, *P* < 0.05).

We next examined the occurrence of miRNAs carrying MNRs of 4 repeats or longer. In expression-unweighted analysis, the frequencies of miRNAs containing MNR(s) ranged from 3.77–4.46%, 6.48–7.84%, 5.16–5.69%, and 5.12–6.26% for adenine, uracil, guanine, and cytosine, respectively (Fig 6A). Among them, adenine repeat-containing miRNAs occurred least frequently (*P* < 0.05). Uracil repeat-containing miRNAs exhibited the highest frequencies in muscle and liver tissue miRNAs (*P* < 0.05).

In the expression-weighted analysis, adenine and cytosine repeat-containing miRNAs occurred with frequencies of 10.5% and 10.3% in plasma miRNAs, respectively (Fig 6B). The adenine repeat frequency was 2.70-fold higher than that of the expression-unweighted analysis (*P* < 0.05), while there was no significant difference from the equivalent expression-unweighted analysis for the cytosine repeat frequency. The frequencies for guanine and uracil repeat-containing miRNAs in plasma miRNAs and for any type of MNR-containing miRNAs in non-plasma miRNAs were 2.35% or less under the expression-weighted criterion. These were at least 58.6% lower than the equivalents in the expression-unweight criterion (*P* < 0.05).

## Discussion

The genes for the 366 unique plasma miRNA species identified in the present study are located on all but one of the horse chromosomes (chromosome 31; Fig 1). Interestingly, of the 690 miRNA genes currently identified, none is located on chromosome 31 (http://www.mirbase.org), suggesting that chromosome 31 contributes little or not at all to miRNA expression.

Clustering analysis showed that the plasma miRNA expression pattern could be readily distinguished from that of other tissues (Fig 2). The potential targets of plasma miRNAs are involved in catalytic, binding, and receptor pathways, as well as metabolic and cellular processes (S1 Fig). Among the 10 most abundant plasma miRNAs of the horse (Table 2), the human orthologs of eca-miR-486-5p, -92a, and -21 also belong to the 10 most abundant miRNAs in human plasma [20], which indicates their importance in mammalian plasma.

Consistent with previous studies in humans and animals [9–13], uracil and guanine were the most frequent nucleotides in tissue miRNAs in the expression-unweighted criterion (Fig 4). This was also observed in the expression-weighted criterion except for the muscle, in which adenine was the predominant nucleotide. The eca-miR-1 species with its high adenine content (nine of 22 nucleotides) accounted for 86.4% of the total miRNA reads in the muscle (S4 Table), and may be responsible for the high adenine content in the expression-weighted analysis. Uracil was the most frequent nucleotide identified at both ends of miRNAs where guanine and cytosine were under-represented (Fig 5); this has previously been reported [9, 13], suggesting that similarities exist between the nucleotide composition of horse miRNAs and those of other animals.

Cytosine was the least frequent nucleotide in the miRNAs of all tissues analyzed, but its under-representation was greater in non-plasma tissues than the plasma (Fig 4). Indeed, analysis of the position-dependent nucleotide composition found that most nucleotide positions in plasma miRNAs had higher cytosine contents than those of other tissues (Fig 5). These findings suggest that cytosine is non-preferentially included in many horse tissue miRNAs, but is less under-represented in plasma miRNAs in a generally position-independent manner. However, the overall guanine–cytosine content of plasma miRNAs, which may augment the strength of base-pairing to mRNA targets [14], was slightly higher than or similar to other tissue miRNAs because the high cytosine content tended to be compensated for by low guanine content (Fig 4). In contrast to the plasma miRNAs, cytosine frequencies were lower in the expression-weighted analysis than the -unweighted analysis in non-plasma tissues, suggesting that miRNA species with high expression levels tend to have lower cytosine contents.

Despite differences in the position-dependent cytosine content among miRNAs of the four tissues (Fig 5), the patterns of position-dependent cytosine frequencies were comparable among the tissue miRNAs in the expression-unweighted criterion, possibly reflecting common miRNA structures. The CVs for cytosine frequencies were similar among the four tissue miRNAs, ranging from 15.9 to 17.6%. Additionally, the lowest and highest cytosine contents were observed at nucleotide positions 3′1 and 5′3/5′4, respectively, in the four tissue miRNAs under the expression-unweighted criterion.

A small number of miRNA species accounts for the majority of the total read counts in many tissues [22, 36–38], indicating that rigorous selection must occur to use only specific miRNAs. This trend was also observed in horse tissues, but to a lower extent in the plasma compared with other tissues (Table 2 and S4 Table). The high CVs for position-dependent nucleotide frequencies in the expression-weighted criterion compared with the -unweighted criterion might reflect the tissue-dependent over-representation of a small number of miRNA species.

The patterns for MNR-containing miRNA occurrence frequencies were generally similar among the four tissue miRNAs in the expression-unweighted criterion (Fig 6A), possibly reflecting the common structure of miRNAs. A 22-nt ssRNA could have 4^22^ possible sequence combinations, of which about 5.57% could have MNR(s) of at least four repeats for each nucleotide. The present study showed that the occurrence frequencies for guanine and cytosine repeat-containing miRNAs were similar to the predicted level and that adenine and uracil repeat-containing miRNAs were under- and over-represented, respectively. In the expression-weighted criterion, however, the MNR occurrence frequencies were much lower than the predicted level, except for adenine and cytosine repeat-containing miRNAs in the plasma. These findings suggest that MNR-containing miRNAs tend to be expressed in a non-preferential manner. The strikingly high expression levels of adenine and cytosine repeat-containing miRNAs in the plasma suggest a preferred expression of miRNAs containing these repeats.

## Conclusion

In view of their location, plasma miRNAs may have distinctive structural characteristics and functional roles. In support of this hypothesis, plasma miRNAs exhibited a unique nucleotide composition with respect to cytosine content and that of adenine and cytosine repeats. Because plasma is unlikely to produce miRNAs, the origin (release) and fate (stability) of plasma miRNA species are responsible for determining the nucleotide composition. It can be hypothesized that structural characteristics are required for miRNA biological functions, but further studies are necessary to examine the sources, functions, and trafficking of miRNAs circulating in the blood plasma.

## Supporting Information

S1 Fig Gene ontology of the predicted target genes for the 10 most highly expressed miRNA species in horse plasma. Categorization of miRNA-target genes was performed according to biological processes (A) and molecular functions (B).
(PDF)

S1 Material A Perl script used to estimate the number of 22-nt miRNAs containing at least one MNR (four nucleotides or longer) for each nucleotide. (PL)

S1 Table Five most abundant putative novel miRNA species in horse plasma. Location represents the chromosome on which the miRNA genes are located, start and end sites, and either sense [+] or antisense strand [−].
(PDF)

S2 Table MiRNA species that showed higher levels of expression (CPM) in the plasma compared with the liver, colon, and muscle tissues. (PDF)

S3 Table MiRNA species that showed lower levels of expression (CPM) in the plasma compared with the liver, colon, and muscle tissues. (PDF)

S4 Table Ten most abundant miRNA species in the horse colon, muscle, and liver. (PDF)
