# Supplementary material for: Expression of microRNAs in Horse Plasma and Their Characteristic Nucleotide Composition
Source: PLoS One. 2016 Jan 5;11(1):e0146374. doi: 10.1371/journal.pone.0146374 (PMC4711666; doi:10.1371/journal.pone.0146374)

S1 Fig. Gene ontology of the predicted target genes for the 10 most highly expressed miRNA species in horse plasma. Categorization of miRNA-target genes was performed according to biological processes (A) and molecular functions (B).

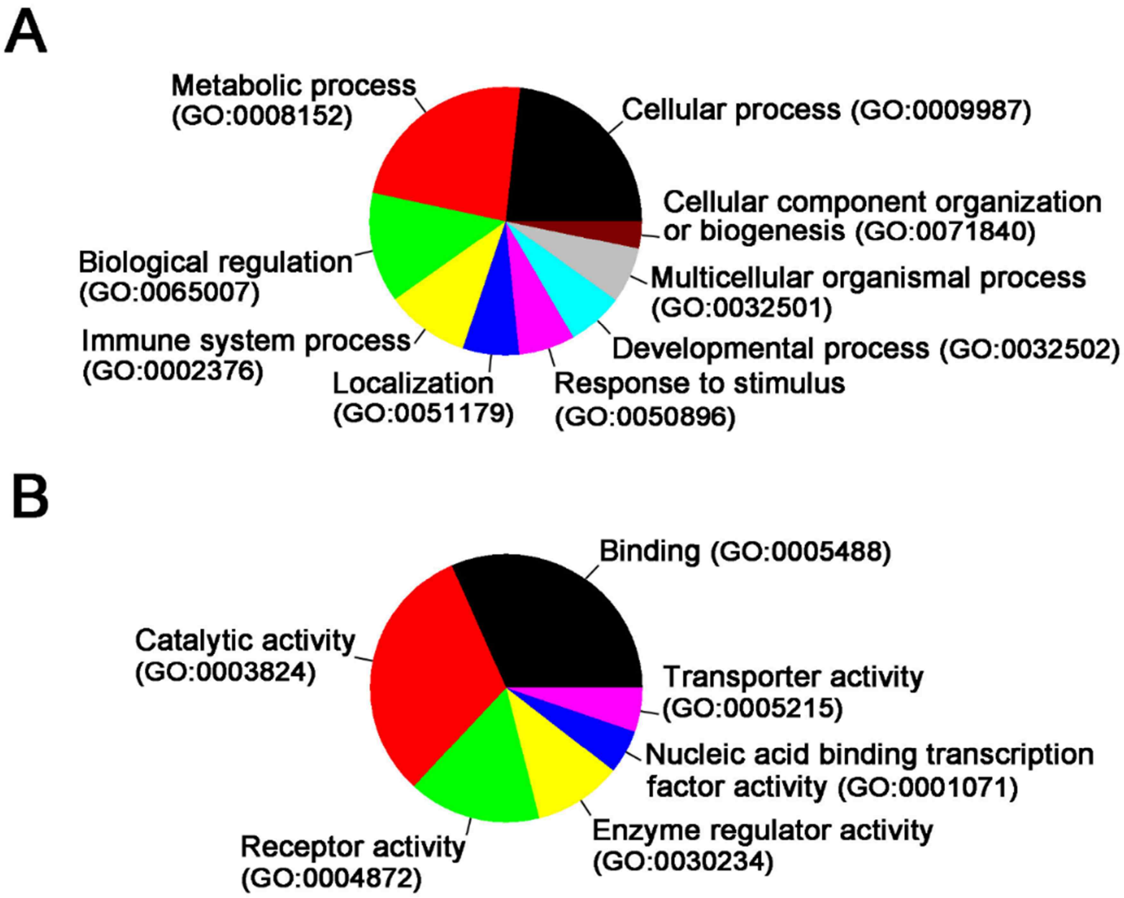

Supplement: S1 Fig — Categorization of miRNA-target genes was performed according to biological processes (A) and molecular functions (B). (PDF) [file pone.0146374.s001.pdf]
