# Supplementary material for: Expression of microRNAs in Horse Plasma and Their Characteristic Nucleotide Composition
Source: PLoS One. 2016 Jan 5;11(1):e0146374. doi: 10.1371/journal.pone.0146374 (PMC4711666; doi:10.1371/journal.pone.0146374)
Supplement: S1 Table — Location represents the chromosome on which the miRNA genes are located, start and end sites, and either sense [+] or antisense strand [−]. (PDF) [file pone.0146374.s003.pdf]

S1 Table. Five most abundant putative novel miRNA species in horse plasma. Location represents the chromosome on which the miRNA genes are located, start and end sites, and either sense [+] or antisense strand [−].

miRNA	Mature sequence	Location
eca-n01	CUGUGCGUGUGACAGCGGCUA	Chr4: 55715268-55715285 [-]
eca-n02	CAGCUGAACCCUACCAGCGCCAGA	Chr11: 19962503-19962590 [-]
eca-n03	AAAAGCUGGGUUGAGAGGGCGA	Chr18: 8712124-8712144 [-]
eca-n04	CACGCUCAUGCACACACCCACA	Chr13: 42256477-42256499 [+]
eca-n05	UCAGUGCAUGACAGAACUUGG	Chr3: 13352618-13352634 [-]
