# Supplementary material for: Expression of microRNAs in Horse Plasma and Their Characteristic Nucleotide Composition
Source: PLoS One. 2016 Jan 5;11(1):e0146374. doi: 10.1371/journal.pone.0146374 (PMC4711666; doi:10.1371/journal.pone.0146374)
Supplement: S2 Table — (PDF) [file pone.0146374.s004.pdf]

S2 Table. MiRNA species that showed higher levels of expression (CPM) in the plasma compared with the liver, colon, and muscle tissues.

miRNA	plasma #1 (SRX170338)	plasma #2 (SRX170339)	plasma #3 (SRX170340)	colon #1 (SRX187171)	colon #2 (SRX187172)	colon #3 (SRX187173)	colon #4 (SRX187174)	muscle #1 (SRX187166)	muscle #2 (SRX187167)	muscle #3 (SRX187168)	muscle #4 (SRX187169)	liver #1 (SRX187162)	liver #2 (SRX187163)	liver #3 (SRX187164)	liver #4 (SRX187165)
mir-93	2008.82	1290.66	2003.21	149.15	77.97	89.26	114.39	48.82	31.96	52.75	53.36	90.59	65.26	55.57	91.91
mir-92b	188.42	118.84	120.43	21.86	24.39	38.62	17.48	13.12	3.20	3.00	13.12	6.66	10.73	5.53	6.51
mir-92a	11326.10	4507.78	6756.36	773.10	848.84	724.08	358.88	517.01	694.30	819.99	1058.18	1737.68	1490.07	1219.54	977.53
mir-9180	1.32	2.10	3.85	0.13	0	0	0.05	0.10	0	0	0.58	0	0	0.35	0
mir-9177	12.15	3.80	7.10	0.06	0	0	0	0	0	0	0	0	0	0.24	0
mir-9159	26.64	10.32	46.02	1.51	0.92	2.27	0.55	1.14	0.43	0	0.87	0.34	1.25	0.47	0.95
mir-9097	33.78	35.69	10.31	1.26	4.36	4.26	1.81	1.04	0.64	1.50	1.46	5.97	3.07	2.94	2.61
mir-9060	78.72	10.83	36.24	5.40	3.38	4.48	5.33	5.20	8.10	10.25	12.25	10.22	7.95	4.95	6.16
mir-9021	22.58	28.26	11.57	3.02	9.34	7.75	3.57	2.39	6.18	9.00	9.62	3.21	3.07	2.12	2.84
mir-8992	15.29	12.45	6.22	1.63	4.06	1.27	1.04	1.04	0.85	1.50	2.62	0.23	0.10	0.12	0.47
mir-8986a	2.41	2.86	2.45	0.25	0.92	0.89	0.49	0.10	1.28	0.75	0.58	0.23	0.19	0	0.36
mir-8946	8.60	4.20	5.93	0.75	1.17	1.49	0.88	0.42	1.07	0.75	0	0.23	0.58	0.71	0.71
mir-8917	1.44	2.10	2.55	0.06	0.31	0.28	0.11	0.0	0.21	0.25	0	0.34	0	0.59	0
mir-744	566.38	565.46	283.09	161.03	234.65	271.54	147.86	109.40	199.41	113.25	151.92	212.06	98.52	106.44	111.22
mir-7177b	12.44	7.29	5.05	0.94	3.20	1.83	1.26	0.83	1.07	1.00	3.21	0.57	0.86	0	0.95
mir-664	13.60	9.47	30.73	0.44	1.47	1.27	1.21	7.70	1.07	1.25	0.29	0.57	0.19	0.35	0.36
mir-652	175.40	100.75	169.62	22.81	12.29	3.54	2.69	3.64	2.77	4.50	4.67	1.84	1.53	1.18	3.79
mir-628a	29.20	12.45	30.31	1.38	2.27	1.49	4.01	4.58	4.69	6.00	8.46	2.41	2.49	1.65	2.13
mir-551a	6.50	3.69	11.39	0.31	1.23	0.55	1.37	0.42	0.21	0.50	0	0.69	0.67	1.06	0.24
mir-532-5p	62.65	58.31	78.86	41.15	28.02	47.20	22.87	30.19	45.38	33.50	25.95	33.75	24.73	26.26	28.07
mir-505	74.06	54.37	82.65	5.09	6.14	4.37	2.58	3.43	1.92	4.75	2.62	12.63	10.73	6.71	9.71
mir-491-5p	3.47	1.76	2.57	0.19	0.31	0.17	0.27	0.62	0.64	0.25	0.87	0.11	0.19	0.24	0
mir-486-5p	26169.85	5633.45	14718.36	35.31	85.41	60.32	128.13	4274.23	2946.77	2630.47	3574.03	362.35	402.23	204.98	108.61
mir-451	1263.55	1077.84	1256.46	57.11	46.14	18.48	99.11	677.83	349.39	569.24	799.83	473.60	650.16	321.07	209.06
mir-425	248.35	116.18	266.85	2.58	1.72	2.38	1.98	1.77	1.28	1.00	1.17	1.49	1.82	1.06	2.37
mir-423-3p	412.79	193.94	212.99	62.70	123.56	142.21	72.23	64.02	60.50	59.25	78.73	76.35	47.06	56.28	46.90
mir-383	87.35	4.37	65.63	5.09	11.43	12.62	7.42	0.10	0.21	1.75	0	8.50	0.29	22.96	8.41
mir-374b	153.42	61.63	453.64	2.39	4.12	5.04	9.34	8.95	2.13	2.25	2.92	3.33	4.03	1.53	2.96
mir-363	44.74	34.98	57.28	11.18	4.79	7.53	7.86	2.39	1.70	3.00	1.17	2.07	3.16	1.18	1.78
mir-361-5p	1205.75	483.16	2149.42	16.46	10.63	7.14	9.95	9.26	4.90	6.00	5.54	7.23	6.80	5.65	9.12
mir-361-3p	20.94	37.14	22.95	4.96	4.61	7.42	3.46	1.04	1.49	2.75	3.21	2.41	3.16	2.71	2.49
mir-3613	12.59	13.30	18.04	1.01	0.98	0.72	0.60	0.94	1.49	0.75	1.46	0.34	0.19	0.47	0.95
mir-350	37.58	19.84	43.01	5.28	0.31	0.39	1.37	13.12	14.91	7.75	11.37	0.69	0.29	0.59	0.71
mir-345-3p	108.46	68.18	76.49	8.86	11.43	8.36	11.98	20.30	12.78	25.25	25.08	7.00	3.64	3.41	6.28
mir-342-5p	25.44	57.72	17.84	4.15	18.13	14.72	6.32	2.81	1.70	1.75	0.58	4.02	1.92	2.47	3.55
mir-342-3p	3837.22	1024.46	2723.26	61.51	311.09	265.45	209.98	21.96	13.42	27.50	16.62	13.09	9.30	8.01	15.99
mir-331	789.57	717.47	180.83	3.33	13.64	4.54	10.44	78.69	31.74	36.00	35.87	3.56	10.54	3.53	12.44
mir-328	263.08	48.90	221.66	1.32	7.13	2.43	3.24	1.98	1.07	0.50	0.58	0.57	0.38	0.94	0.83
mir-326	18.41	7.06	24.83	0.19	0.49	0.28	0.22	0.42	0.64	0	0.29	0	0.29	0.12	0.24
mir-324-5p	22.88	12.59	16.74	5.72	3.26	1.27	4.56	7.60	1.28	2.25	1.17	1.15	1.34	2.12	1.42
mir-324-3p	27.34	9.33	14.33	1.51	2.70	0.94	1.32	1.56	1.07	0.25	1.75	0.57	0.67	0.59	0.59
mir-3200	71.40	35.32	85.37	0.88	0.74	1.00	0.60	1.14	0.43	0.50	0.58	0.11	0	0	0.12
mir-27a	1020.05	848.64	1106.44	384.45	771.79	606.43	898.50	226.50	413.51	351.00	443.22	119.06	243.71	103.61	133.49
mir-26a	4165.51	5738.76	8417.05	1042.95	1619.09	1372.62	2149.35	2445.58	1731.59	2894.71	3055.00	2553.76	2371.67	2088.81	2078.12

mir-25	5748.72	4475.26	4653.75	442.31	539.29	568.53	464.31	196.83	257.57	307.25	287.51	325.95	383.25	171.90	197.21
mir-2483	60.19	38.27	31.08	0	0	0	0.05	0.31	0.21	0.50	0	0.11	0.10	0	0
mir-24	12120.09	2742.55	10010.86	610.63	948.93	745.33	1111.72	420.21	623.35	574.24	894.60	220.78	213.04	181.91	199.94
mir-23a	7594.78	3692.93	7087.42	700.03	445.95	397.15	467.61	705.52	274.18	335.50	378.78	68.08	96.03	75.00	103.40
mir-223	8354.10	4147.49	11687.57	21.36	4.73	9.13	6.21	18.84	2.34	2.50	3.50	10.91	11.02	7.30	11.25
mir-222	5492.18	4170.42	2009.49	1662.69	1161.33	2539.05	660.22	86.81	371.75	289.50	282.55	782.33	765.06	722.80	780.43
mir-221	4258.98	4694.68	3964.92	970.57	694.24	1407.37	731.07	120.85	269.92	228.00	294.80	738.47	561.70	804.75	683.19
mir-211	64.61	5.87	38.01	2.58	1.23	1.22	1.04	2.91	0	0.75	0	0.46	0.58	1.30	1.18
mir-21	6250.30	4974.95	11741.72	3605.91	585.98	801.77	2676.71	1197.86	1760.56	3396.71	2968.98	2314.73	2500.57	2136.02	2056.44
mir-20b	2.87	1.08	4.74	0.88	0.25	0.28	0.66	0.52	0	0.25	0	0.11	0	0	0
mir-19b	36.71	25.32	113.67	14.39	1.60	1.05	9.89	6.35	2.56	11.00	2.33	13.43	11.21	12.24	8.41
mir-197	162.09	154.50	186.02	6.16	7.13	5.70	5.44	6.14	2.56	5.00	6.12	1.26	1.25	1.88	3.32
mir-196b	29.39	18.31	51.33	24.63	4.24	5.31	11.76	5.00	3.83	5.00	3.21	0	0	0.24	0
mir-191a	40517.88	20994.61	21035.54	2582.31	1947.99	3267.17	1474.01	1218.26	790.16	1313.98	1580.13	2436.66	2342.73	2019.70	3320.37
mir-18a	10.73	6.07	14.20	1.51	0.18	0	1.10	0.10	0	0	0	0.34	0.10	0	0.47
mir-187	8.69	6.15	6.16	1.51	1.72	0.72	0.99	0.31	0.85	0.75	0.87	1.15	2.30	0.59	1.18
mir-186	255.63	322.19	562.86	41.78	39.75	43.00	56.18	51.73	34.51	60.50	61.53	57.18	48.30	48.04	49.98
mir-185	78.06	46.66	15.18	7.16	22.30	6.03	15.67	4.06	10.23	7.00	7.29	2.87	11.50	2.00	6.99
mir-1839	5149.84	2224.72	5189.88	745.15	502.24	575.72	1005.03	332.57	671.29	543.49	658.41	920.10	814.42	781.67	755.92
mir-181b	581.47	466.55	621.72	120.82	174.68	202.64	235.87	346.20	175.76	153.50	124.80	130.31	92.48	90.42	56.14
mir-17	123.38	109.57	341.53	36.82	6.64	6.53	14.40	7.18	3.62	9.25	7.29	13.89	11.98	12.83	10.42
mir-16	1168.63	643.53	2015.24	364.34	132.23	76.25	121.20	128.97	119.73	188.50	146.96	106.20	80.31	84.07	84.93
mir-15b	469.04	596.47	570.82	66.41	41.23	27.45	13.52	11.14	6.39	10.25	6.42	7.46	8.34	10.36	9.00
mir-15a	84.94	57.89	126.74	55.60	11.55	6.86	17.42	33.20	29.61	45.50	22.45	14.12	12.27	14.01	11.49
mir-155	120.74	129.67	135.31	47.18	27.28	40.78	45.51	8.54	9.16	11.00	9.62	13.09	8.15	6.48	4.74
mir-150	779.98	1154.58	1340.16	27.20	6.21	26.12	17.37	62.04	13.85	20.25	25.66	2.64	3.74	1.18	4.74
mir-146b-5p	125.42	69.14	202.63	54.10	45.71	59.65	66.24	19.57	13.21	30.00	18.95	40.64	41.69	41.56	17.53
mir-146a	329.80	245.48	669.75	73.89	19.78	33.37	73.71	15.41	9.16	10.50	9.33	67.05	46.77	40.03	18.00
mir-142-5p	1636.08	1218.65	1534.58	92.73	43.19	76.81	156.55	38.62	28.76	92.50	86.02	135.02	145.19	87.13	117.85
mir-142-3p	102.15	41.08	284.73	20.54	0.25	0.50	7.64	1.77	0.21	3.50	2.04	2.76	4.03	3.06	5.80
mir-140-5p	3.40	3.66	10.80	1.26	0.18	0.28	1.10	1.46	0.43	0.75	1.75	0.46	0.48	0.12	0.83
mir-1379	33.02	17.04	15.56	4.59	4.24	2.66	3.52	8.43	4.26	3.25	6.12	2.53	1.82	0.82	2.96
mir-130b	85.02	32.52	95.91	10.18	2.64	4.87	3.30	1.14	0.85	2.75	0.58	1.26	1.15	0.47	0.12
mir-1307	1486.55	2218.20	971.83	173.09	339.53	294.72	252.47	83.58	93.74	186.00	248.14	138.46	30.38	104.20	95.11
mir-1301	121.32	49.27	70.92	3.96	9.22	9.79	3.46	1.67	2.34	3.00	2.62	4.02	2.01	2.47	2.84
mir-129a-3p	1.51	4.62	3.34	0.13	0.43	0.39	0.16	0.21	0	0	0	0	0.10	0	0.12
mir-1271b	4.38	7.60	5.15	0.75	0.31	0.50	0.27	0	0	0.50	0.29	0.11	0.29	0.12	0.24
mir-1180	8.15	1.22	6.28	1.38	2.58	0.66	1.04	0.31	0.85	1.25	1.17	0.34	0.38	0.12	0.71
mir-107a	6313.71	6183.51	7390.30	4148.81	3654.26	4377.22	5288.94	1714.46	2102.70	2065.97	2134.16	5573.67	4711.71	4542.37	4052.36
mir-106b	172.64	120.09	259.71	60.82	13.46	12.45	67.28	15.30	20.24	30.00	23.33	26.18	25.01	24.49	23.10
