# Supplementary material for: Expression of microRNAs in Horse Plasma and Their Characteristic Nucleotide Composition
Source: PLoS One. 2016 Jan 5;11(1):e0146374. doi: 10.1371/journal.pone.0146374 (PMC4711666; doi:10.1371/journal.pone.0146374)
Supplement: S3 Table — (PDF) [file pone.0146374.s005.pdf]

S3 Table. MiRNA species that showed lower levels of expression (CPM) in the plasma compared with the liver, colon, and muscle tissues.

miRNA	plasma #1 (SRX170338)	plasma #2 (SRX170339)	plasma #3 (SRX170340)	colon #1 (SRX187171)	colon #2 (SRX187172)	colon #3 (SRX187173)	colon #4 (SRX187174)	muscle #1 (SRX187166)	muscle #2 (SRX187167)	muscle #3 (SRX187168)	muscle #4 (SRX187169)	liver #1 (SRX187162)	liver #2 (SRX187163)	liver #3 (SRX187164)	liver #4 (SRX187165)
mir-497	0.45	0.31	0.31	115.10	74.35	55.67	107.24	62.56	73.50	56.75	63.28	45.12	55.78	57.57	43.94
mir-199a-5p	2.11	0.34	1.00	241.32	221.07	275.35	316.67	135.94	240.52	138.50	186.62	216.31	308.69	271.15	173.88
mir-199a-3p	382.39	185.86	360.49	7625.30	7670.92	7783.67	11484.18	2519.38	8989.21	4640.44	5062.61	7277.02	9057.14	8418.58	5086.03
mir-195	0.85	0.79	1.23	206.20	133.64	71.55	152.65	89.41	74.56	83.00	105.56	75.55	76.67	91.48	73.55
let-7a	29559.12	64477.76	9747.78	117137.10	249016.85	260193.23	217645.80	62406.24	196103.29	163391.16	200088.67	247534.44	199096.06	170958.16	148777.89
