# Supplementary material for: Expression of microRNAs in Horse Plasma and Their Characteristic Nucleotide Composition
Source: PLoS One. 2016 Jan 5;11(1):e0146374. doi: 10.1371/journal.pone.0146374 (PMC4711666; doi:10.1371/journal.pone.0146374)
Supplement: S4 Table — (PDF) [file pone.0146374.s006.pdf]

S4 Table. Ten most abundant miRNA species in the horse colon, muscle, and liver.

miRNA	CPM per total CPM (%)		
	Colon	Muscle	Liver
eca-let-7a	37.6	4.26	12.7
eca-let-7c	9.25	0.73	3.46
eca-let-7e	3.71	0.18	1.23
eca-let-7f	16.1	3.13	8.06
eca-let-7g	2.56	0.41	1.22
eca-miR-1	5.01	86.4	-
eca-miR-29a	-	0.18	-
eca-miR-101	-	0.27	1.18
eca-miR-122	-	-	65.5
eca-miR-143	5.31	-	-
eca-miR-192	5.00	-	2.13
eca-miR-199a-3p	1.59	-	0.50
eca-miR-206	-	1.46	-
eca-miR-378	-	0.59	-
eca-miR-423-5p	1.65	-	0.51
Total	87.8	97.6	96.5
